# Gendered associations of situational and dispositional factors with exclusion from social relations and loneliness in older age

**DOI:** 10.3389/fpubh.2025.1445662

**Published:** 2025-02-10

**Authors:** Georgios Pavlidis

**Affiliations:** ^1^Institute of Social and Psychological Studies, Karlstad University, Karlstad, Sweden; ^2^Division of Ageing and Social Change, Institute of Culture and Society, Linkoping University, Norrkoping, Sweden

**Keywords:** personality, exclusion from social relations, aging, loneliness, gender

## Abstract

**Background:**

States of exclusion from social relations (ESR) refers to severe social isolation in older age that is not always typified by increased loneliness. Relevant deficiencies in the social network of older persons may be gendered and associated with personality and socioeconomic barriers, with direct implications for older persons’ welfare. Although the contribution of personality traits and socioeconomic barriers in shaping ESR states in older age are often debated, empirical evidence that addresses their unique contribution is limited. Therefore, the aim of this study was to examine the gender-stratified associations of situational (e.g., marital status, socioeconomic conditions) and dispositional factors (i.e., personality traits) with ESR states and loneliness in older age.

**Methods:**

A cross-sectional and gender-stratified secondary analysis of a sample (*N* = 36,814) from the Survey on Health, Aging, and Retirement in Europe was conducted using logistic regression models.

**Results:**

The probability of ESR was higher among older men. Certain situational factors (e.g., widowed, never married) significantly increased the probabilities of ESR for both genders, while other (e.g., divorce) had a gender-specific significance. Less extraversion among older women and less conscientiousness among older men was associated with an increased probability of ESR in later life. Within ESR states, older men living alone and older women who are less extraverted were more at-risk of loneliness.

**Conclusion:**

Situational factors are more predictive of ESR states than personality traits, yet a gendered perspective is needed when assessing the risk factors of ESR and loneliness in later life.

## 1 Introduction

Loneliness and social isolation in older age are societal challenges and major public health concerns that challenge the cohesion of modern societies (1, 2). While loneliness and social isolation are often conflated in public discourses (3), these constructs describe related, yet distinct, aspects of relational deficits in older age. Loneliness is defined as the subjective perception of social isolation, stemming from having fewer social relations than desired, or from an unfulfilled intimacy with established social relations (4, 5). Discordantly, social isolation refers to objective shortages in functional (e.g., receiving support) and structural (e.g., in social network size) aspects that can typify the social network of older persons (6).

Loneliness and social isolation are constructs that are weakly to moderately correlated in quantitative studies (7), often explained from the thesis that some socially isolated older person may not feel lonely, while others may feel lonely “in the crowd” (4). Relevant studies (8, 9) showcase dissimilar levels of psychological distress among older persons who are challenged by different conjunctions of social isolation and loneliness (i.e., lonely *and* socially isolated; lonely *but not* socially isolated; *not* lonely *but* socially isolated). For example, Menec et al. (8) analyzed data from the Canadian Longitudinal Study of Aging (CLSA) and found that older persons who are *socially isolated and lonely* are more psychologically distressed than those who are *socially isolated but not lonely*; while older persons who are *lonely but not socially isolated* are more psychologically distressed than those who are *socially isolated but not lonely*. Most older people are *neither socially isolated nor lonely* and as a group tend to have the lowest levels of psychological distress.

Exclusion from social relations (ESR) is a more recent and kindred concept to social isolation, defined as a state of being socially and emotionally disconnected from meaningful relationships and social opportunities (10). It has been argued, however, that scoring zero in some objective measure of social network size constitutes a distinct ESR state that is qualitatively different from that of having a very small network size (11–13). Pavlidis et al. (14) used this concept to argue that ESR in older age refers to social isolation at the extreme, even when these exclusionary states are not always perceived negatively by the older “network-less” persons themselves. Therefore, ESR states may not always have negative consequences for the wellbeing of older persons. Accordingly, Pavlidis et al. (14) found that more than half of older persons in ESR states (i.e., those who do not have someone to talk about important issues with, or any other person who is important for them for any other reason) are moderately to highly satisfied with their solitary state. In the same study, older persons who are content with being in ESR states report high levels of quality of life (QoL), showing no statistically significant differences in this respect with older persons who are embedded in a social network and are satisfied with their social relations.

These findings could be explained through the lens of positive solitude, which refers to the positive aspects of solitary states, where individuals may volitionally choose to spend time alone (15). For example, Toyoshima and Sato (16) found that among older persons who frequently spent time alone, having little social interaction does not decrease their subjective wellbeing but correlates with positive affect. Lay et al. (17) argued that individuals who desire solitude felt less lonely in their study, and older persons classified as solitude-seekers reported no decrease in positive affect even when they were more likely to spend more time in locations conducive to solitude (e.g., at home). While these paradoxical findings may refer to momentary experiences of solitude, they do contradict the widely held assumption of a universal and innate need for social connectedness (18). Yet, an inclination to solitude has been often attributed to *dispositional factors* such personality traits that predispose individuals to willingly be more socially withdrawn across their lifespan (19, 20). Personality traits are patterns of thoughts, feelings, and behaviors that remain relatively stable across the life course (21). A widely used taxonomy is that of the Big Five personality traits (21), which includes extraversion (describing how energetic, outgoing, and confident a person is), agreeableness (reflecting friendliness, compassion, and a willingness to help), conscientiousness (reflecting one’s desire to be careful and diligent), openness (reflecting the tendency to try new things), and neuroticism (characterized by a tendency to worry, to be temperamental, and to be prone to experiencing negative emotions).

Accordingly, Buecker et al. (22) through meta-analyses found that higher extraversion, agreeableness, conscientiousness, and openness to experience are negatively related with loneliness, whereas higher neuroticism is positively related with loneliness. However, these associations may not be uniformly evident across all age groups; Butkovic et al. (23) found that agreeableness and conscientiousness may be more strongly related to loneliness among older adults than among adolescents. In terms of ESR in older age and its association with personality traits, the evidence is mixed. Litwin and Levinsky (24) found a positive but weak association between extraversion, openness, and agreeableness with the network size of older Europeans. However, Schutter et al. (20) found that the Big Five personality traits are associated with loneliness but not with social network size among older people.

Besides personality, various *situational factors* may constitute, or even bring older persons to ESR states. Hooker and McAdams (25) argued that (p. 296) *“personality is arguably the driving force behind all antecedents of successful aging, except of course the structural ones.”* Personality traits may shape older persons’ coping behaviors when challenged by significant life events or exclusionary states, but one can hardly argue against in terms of ESR and loneliness, the social context shapes a frame of restrictions when it comes to coping. This tension between dispositional and situational factors, however, and their contribution to ESR and loneliness in older age has been rarely discussed or empirically examined. De Jong-Gierveld (26) argued that loneliness stems from the subjective evaluation of relational deficits and therefore maybe be conceptually distant to situational factors of ESR in older age. Still, over three decades or research indicates that partner status (e.g., being widowed, divorced, or never married), certain living arrangements (e.g., living alone), and ill health (both physical and mental) are situational factors that are associated with loneliness (20, 24, 27–29). Similarly, an increased engagement with the community and other social activities may help older persons in ESR states to compensate relational deficits in their immediate social network, and by that to feel less lonely (30–32). Yet, there is evidence that older persons living alone and rarely visited by friends and family have a 77% increased mortality risk (33).

While situational factors in older age may shape ESR states and co-exist with feelings of loneliness, there are gender differences in the distribution of the relevant risks. Older women are more likely than older men to report network growth in older age (34) and more willing to participate in social activities (35). Yet, older women are more likely than older men to be widowed (49) and experience more difficulties in re-partnering after a divorce, relationship dissolvement, or widowhood (36, 37). Schutter et al. (20) also found gender differences in in the associations between personality traits and loneliness, as higher neuroticism and lower extraversion was associated with loneliness among older women but not among older men. This is hardly surprising, since there is a gendered differentiation on neuroticism and agreeableness across the lifespan, with women scoring higher than men in both personality traits (38).

**Figure 1:**
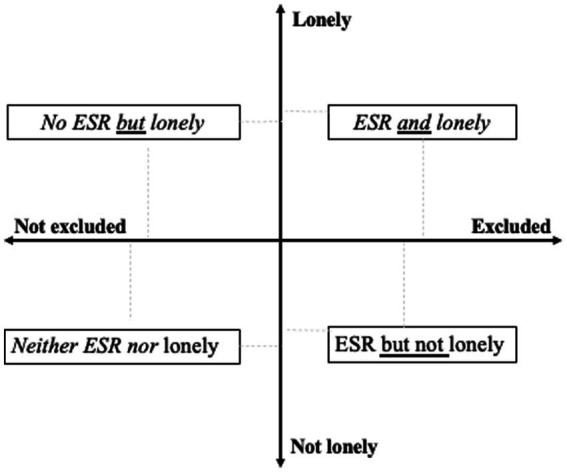
States of exclusion from social relations (ESR) in older age based on measures of social isolation and loneliness. ESR, states of exclusion from social relations, namely scoring zero in network size; lonely are those who feel lonely sometimes or all the time, not lonely those who never felt lonely.

Adding to the limited research that examines public health concerns at the crossroads of ESR and loneliness in older age (8, 9, 12), the aim of this study was to examine the associations of dispositional and situational factors with ESR states that are typified (or not) by loneliness (i.e., ESR and lonely, ESR and not lonely, not ESR but lonely; see Figure 1). The unique contribution of the study lies in the empirical analysis of ESR states in older age within the framework of the wide and lasting debate on the structure-versus-agency contributions to this vulnerability. Based on previous research (11, 14), ESR is conceptualized in this study as social isolation at the extreme, namely not having someone to talk to about important issues, or any other person who is important for any other reason. Socialization in “third places,” namely those interactions in activities outside the home or work (e.g., church, clubs, organizations), was defined in this study as social participation (39). It was hypothesized that over and above social participation, as well as physical and mental health:

- There are gender differences in the associations of situational factors and personality traits with the probability of being in objective ESR states in older age.
- Higher neuroticism and lower extraversion in women, and lower agreeableness in both genders will be associated with higher levels of loneliness among older persons that are challenged by ESR states.

## 2 Materials and methods

### 2.1 Data

A pooled sample from three waves of the Survey on Health, Aging and Retirement in Europe (SHARE) was used (40). The Big Five personality traits inventory was administered for the first time during the seventh wave of SHARE (collected in 2017). The fourth and the sixth waves (collected in 2011 and 2015, respectively) and the eighth wave of SHARE (collected in 2020) have a detailed module inquiring into older persons’ social networks. However, the eighth wave of the SHARE study was not included in this study, since the probably of the data reflecting restrictions in the sociability of older persons posed by the COVID-19 pandemic was significant. For those who participated in both the fourth and the sixth waves of SHARE, data were extracted only from the fourth wave, so no participant was represented twice in the sample. Approximately 54% of the sample came from the fourth wave, and 46% came from the sixth wave.

The sample was restricted to non-institutionalized participants who completed all information (no missing values) about depressive symptoms, loneliness, social networks, and social participation, who were administered the Big Five personality traits inventory in the seventh wave or the eight wave of SHARE, and who responded to the social network modules in private (N = 36,814). The respondents originated from Israel and 18 European countries, namely Austria, Germany, Sweden, Spain, Italy, France, Denmark, Greece, Switzerland, Belgium, the Czech Republic, Poland, Luxembourg, Hungary, Portugal, Slovenia, Estonia, and Croatia. The sample consisted of 42.8% male and 57.2% female respondents. Information about the SHARE survey procedures (e.g., sampling methods, data collection methods, response rates) and its ethics approval can be found in Bergmann et al. (41) and on the official website of the survey.^1^ The demographics of the pooled sample in whole and disaggregated by gender are presented in Table 1.

**Table 1:** Descriptive analyses of demographics, EURO-D, R-UCLA scores, as well as health and social network size variables.

	All M (SD)	Men M (SD)	Women M (SD)
Age	64.81 (8.86)	65.12 (8.66)	64.58 (9.00)
Education (Years)	11.04 (4.41)	11.49 (4.50)	10.70 (4.30)
Income^1^ (Household)	3.91 (6.88)	4.16 (6.9)	3.72 (6.48)
Health			
ADL	0.12 (0.53)	0.11, (0.49)	0.13 (0.56)
IADL	0.21 (0.73)	0.14 (0.61)	0.26 (0.81)
Chronic diseases	1.61 (1.48)	1.52 (1.42)	1.68 (1.53)
Mobility limitations	1.32 (1.99)	0.93 (1.65)	1.61, (2.17)
EURO-D	2.28 (2.16)	1.77 (1.85)	2.66 (2.29)
R-UCLA	3.81 (1.28)	3.66 (1.14)	3.92 (1.37)
SoPA (index)	1.63 (2.29)	1.73 (2.35)	1.55 (2.24)
Network size	2.62 (1.59)	2.38 (1.52)	2.79 (1.61)
Personality traits			
Extraversion	3.47 (0.92)	3.45 (0.91)	3.48 (0.92)
Agreeableness	3.71 (0.80)	3.66 (0.81)	3.74 (0.79)
Conscientiousness	4.14 (0.77)	4.10 (0.79)	4.17 (0.76)
Neuroticism	2.65 (1.00)	2.49 (0.96)	2.76 (1.02)
Openness	3.28 (0.95)	3.24 (0.94)	3.30 (0.96)
Job status	(%)	(%)	(%)
Retired	54.6	60.2	50.5
(Self) Employed	28.9	32.3	26.3
Unemployed	3.2	3.5	3.0
Permanently sick	2.6	2.7	2.6
Homemaker	9.1	0.2	15.8
Other	1.5	1.1	1.8
Marital status	(%)	(%)	(%)
Married	70.2	77.8	64.5
Partnership	1.3	1.5	1.2
Married not cohabiting	1.3	1.3	1.2
Never married	5.5	6.3	4.8
Divorced	9.0	7.7	9.9
Widowed	12.8	5.3	18.4
Living alone	20.5	14.3	25.1
ESR	2.5	3.0	2.1
ESR not lonely	53.8^2^	58.3^2^	49.1^2^
Neither ESR nor lonely	60.5^3^	65^3^	57.1^3^

^1Annual income in 10.000 euros. 2The percentages refer to the sample of older persons in ESR. 3The percentages refer to the sample of older persons outside ESR. M, Mean; SD, Standard deviation; ADL, Activities of daily living; IADL, Instrumental activities of daily living; EURO-D, Depressive symptoms; R-UCLA, loneliness scale; SoPA, social participation; ESR, scoring zero in network size.^

### 2.2 Measures

#### 2.2.1 States of exclusion from social relations (ESR)

The operationalization of ESR states was based on the name generating inventory used in SHARE. Participants in SHARE were asked “Over the last 12 months, who are the people with whom you most often discussed important things? These people may include your family members, friends, neighbors, or other acquaintances,” with the instruction to name up to six persons. In addition, respondents were given the opportunity to list an additional person that was important for them for any other reason (40). According to previous research (11, 14), participants who did not report any person in this inventory were coded as being in an objective ESR state. Participants who reported one person or more in their network were coded as being embedded in a social network (and hence were *not* in an ESR state).

#### 2.2.2 Loneliness

Loneliness was measured in SHARE using the short version of the Revised University of California at Los Angeles Loneliness scale (R-UCLA; (42)). The scale consists of three items asking about loneliness indirectly, namely about the frequency of feeling a lack of companionship, being left out, and isolation from others, with three available responses: hardly ever or never, some of the time, and often. The final score is a summation of the three items and has possible values between 3 and 9 (40), with higher values indicating higher levels of loneliness. The Cronbach’s *α* for the loneliness scale in wave four was *a* = 0.782 and in the sixth wave *a* = 0.751, whereas in the aggregated sample it was *a* = 0.747. Similarly to previous research (27), the loneliness scores in the 7–9 range were limited in this sample, and therefore, loneliness was divided into two categories; not lonely (score = 3) and lonely (score range: 4–9).

#### 2.2.3 Social participation

Participants in the SHARE study were asked whether they have done voluntary or charity work; attended an educational or training course; gone to a sport, social, or other kind of club; and whether they took part in a political or community-related organization. They were also asked about the participation frequency in these activities on a Likert scale, with the possible responses of “almost every day,” “almost every week,” “almost every month,” and “less often.” For this study, participants’ responses were reversed scored and summed, so that higher frequency scores represented more social participation (range: 0–16). For participants who reported not taking part in an activity, the value of zero was assigned as the frequency. Since the social participation index reflected a breadth of activities that may not co-exist (i.e., one may participate in volunteering but not in political or community organizations), no Cronbach alpha was calculated for this measure.

#### 2.2.4 Depressive symptoms

The EURO-D 12-item scale was used to assess the existence of depressive symptoms. The scale uses 12 binary yes/no response items inquiring about the presence of depressive symptoms, with a total score between 0 and 12. These items cover depression, pessimism, suicidality, guilt, sleep, interest, irritability, appetite, fatigue, concentration, enjoyment, and tearfulness during the last month. Higher scores in this scale indicate more depressive symptoms (40). Because the EURO-D reflects a breadth of depressive symptoms that may not co-exist, no Cronbach alpha was calculated.

#### 2.2.5 Personality traits

The Big Five personality traits were measured in SHARE using the 10-item version of the Big Five Inventory (BFI-10) introduced by Rammstedt and John (43). The BFI-10 is an abbreviated version of the BFI-44, measuring each personality trait (openness to experience, conscientiousness, extraversion, agreeableness, and neuroticism) with two items rated on a 5-point scale. Higher scores in the BFI-10 indicate a higher agreement with statements that are consistent with a personality trait. The scale construction favored brevity and breadth, as much as this was possible with only two items per trait. According to Levinsky et al. (44), the BFI-10 used in SHARE has a strong congruency between the idealized Big-Five structure (c = 0.94) but poor internal consistency (Spearman-Brown coefficient = Openness *r_Spearman_* = 0.45, Conscientiousness *r_Spearman_* = 0.50, *r_Spearman_* = 0.53, Agreeableness *r_Spearman_* = 0.39, and Neuroticism *r_Spearman_* = 0.67). Given the brevity of the instrument and following previously established research practices (24, 45), these values were seen as acceptable.

#### 2.2.6 Demographics

Two categories were constructed for household size: one representing those living alone, and one for those who were living with one or more persons in the household. Household income was an imputed variable in euros, available in the “gv_imputation” module of the SHARE database. Education represents years of attendance in full-time education.

#### 2.2.7 Health

The number of chronic health conditions (range: 0–12) was an imputed variable available in the gv_health module of the SHARE study, which contains a broad range of physical and mental health conditions. The limitations in activities of daily living (ADL) addresses difficulties in dressing, walking across a room, bathing, eating, getting in or out of bed, and using the toilet (range: 0–6). The limitations in instrumental activities of daily living (IADL) addresses difficulties using a map, preparing a hot meal, shopping for groceries, making telephone calls, taking medications, doing work around the house or garden, and managing money (range: 0–8). In addition, participants were asked whether they have any mobility limitations in 10 activities including arm function and fine motor limitations (range: 0–10). The chronic conditions and limitations under this section were neither weighted (e.g., certain types of limitations were not considered to have greater health-related significance to social isolation), nor was any threshold applied in the analysis. For all variables, higher scores indicate more health problems or more health-related functional difficulties (40).

### 2.3 Statistical analyses

The data were analyzed using SPSS v.28. The *p*-value cutoff was set to *p* < 0.050, and significance testing were two-tailed.

#### 2.3.1 Gendered probability of ESR states

To examine the first hypothesis of the study, a binary logistic model (Model 1) was constructed with being ESR states (versus not being in ESR states) to be the target value. In Model 1, the predictors were gender, age, living alone, education, marital and employment status (effect coding), health variables, depressive symptoms, social participation, and the Big Five personality traits. To examine gender differences in the prediction of ESR states, the same model was used in gender stratified analyses excluding gender as a predictor (Models 2 and 3).

#### 2.3.2 Gendered probability of feeling lonely in ESR states

To examine the second hypothesis of the study, a binary logistic model (Model 4) was constructed with feeling lonely within ESR states (versus not feeling lonely within ESR states) to be the target value. In Model 4 as well, gender, age, living alone, education, marital and employment status (effect coding), health variables, depressive symptoms, social participation, and the Big Five personality traits were the predictors. To examine gender differences in the prediction of loneliness among older persons in ESR states, the same model was used in gender stratified analyses excluding gender as a predictor (Models 5 and 6). In models 4–6, the sample was restricted to those situated in ESR states.

## 3 Results

### 3.1 Gendered probability of ESR states

The binary logistic regression (Model 1) examined the probability of being in ESR states and yielded a statistically significant model [*χ*^2^(26) = 481.818, *p* < 0.001, Nagelkerke *R^2^* = 0.064]. Being male (*OR* = 0.592, *CI:* 0.510–0.688, *p* < 0.001), fewer years of education (*OR* = 0.959, *CI:* 0.944–0.975, *p* < 0.001), living alone (*OR* = 0.502, *CI:* 0.401–0.629, *p* < 0.001), having fewer chronic diseases (*OR* = 0.889, *CI:* 0.841–0.938, *p* < 0.001), more depressive symptoms (*OR* = 1.053, *CI:* 1.018–1.089, *p* = 0.003), less social participation (*OR* = 0.884, *CI:* 0.850–0.918, *p* < 0.001), and being less extraverted (*OR* = 0.874, *CI:* 0.810–0.943, *p* < 0.001) were all statistically significant associated with an increased probability of being in ESR states. Compared to those married and living with their spouse, older persons that were never married (*OR* = 2.217, *CI:* 1.685–2.917, *p* < 0.001), divorced (*OR* = 1.540, *CI:* 1.177–2.017, *p* = 0.002), and widowed (*OR* = 1.554, *CI:* 1.220–2.010, *p* < 0.001) were statistically significantly more likely to be in ESR states. Similarly, compared to those who were employed or self-employed, retired older persons were statistically more likely (*OR* = 0.635, *CI:* 0.513–0.786, *p* < 0.001) to be in ESR states (see Table 2).

**Table 2:** Binary logistic regression analyses (Models 1, 2, 3) predicting being in states of exclusion from social relations (ESR), with demographics, EURO-D, health, social participation, and personality traits as predictors.

	Model 1 (all)		Model 2 (men)		Model 3 (women)	
	B	Exp(B)	B	Exp(B)	B	Exp(B)
Gender (male/female)	−0.524*	0.592	–	–	–	–
Age	0.001	1.001	−0.001	0.999	0.003	1.003
Education	−0.042*	0.959	−0.039*	0.962	−0.043*	0.957
Living alone (yes/no)	−0.688*	0.502	−0.776*	0.460	−0.600*	0.549
Income	0.000	1.000	0.000	1.000	0.000	1.000
Marital status^1^						
Registered partnership	0.006	1.006	−0.019	0.981	0.042	1.043
Married not cohabiting	0.162	1.175	0.439	1.551	−0.474	0.622
Never married	0.796*	2.217	0.826*	2.284	0.691*	1.995
Divorced	0.432*	1.540	0.221	1.247	0.596*	1.815
Widowed	0.441*	1.554	0.556*	1.744	0.373*	1.453
Employment status^2^						
(Self) Employed	−0.455*	0.635	−0.413*	0.662	−0.534*	0.587
Unemployed	−0.180	0.835	−0.121	0.886	−0.302	0.739
Permanently sick	−0.145	0.865	0.001	1.001	−0.294	0.745
Homemaker	−0.181	0.834	−17.8	0.000	−0.221	0.802
Other	0.160	1.174	0.169	1.184	0.146	1.158
ADL	0.108	1.114	0.144	1.155	0.086	1.090
IADL	−0.077	0.926	−0.052	0.950	−0.090	0.914
Chronic diseases	−0.118*	0.889	−0.127*	0.880	−0.107*	0.898
Mobility limitations	−0.032	0.969	−0.055	0.946	−0.024	0.976
EURO-D	0.052*	1.053	0.035	1.035	0.061*	1.063
Social participation	−0.124*	0.884	−0.084*	0.919	−0.177*	0.838
Personality traits						
Extraversion	−0.135*	0.874	−0.047	0.954	−0.224*	0.800
Agreeableness	−0.055	0.946	0.007	1.007	−0.110	0.896
Conscientiousness	−0.057	0.945	−0.124*	0.884	0.007	1.007
Neuroticism	−0.042	0.959	0.022	1.022	−0.097	0.907
Openness	−0.001	0.999	0.012	1.012	−0.012	0.988

^1^Reference category: married and living with spouse; ^2^Reference category: retired. ADL, Activities of daily living; IADL, Instrumental activities of daily living; EURO-D, Depressive symptoms scale. Significance level: *p < 0.050. Statistically significant values at the *p* < 0.050 are in bold.

The same binary logistic regression analysis was repeated separately for men (Model 2) and women (Model 3), yielding statistically significant models for both men [*χ^2^_men_* (25) = 231.373, *p* < 0.001, Nagelkerke *R^2^* = 0.063] and women [*χ^2^_women_* (25) = 257.373, *p* < 0.001, Nagelkerke *R^2^* = 0.066]. Fewer years of education, living alone, never having been married, being widowed, fewer chronic diseases, less social participation, and retirement remained factors with a statistically significant increased probability of being in ESR states for both genders (see Table 2). Notable gender differences emerged in the role of personality traits, with less conscientiousness for men (*OR_men_* = 0.884, *CI:* 0.785–0.995, *p* = 0.041) and less extraversion for women (*OR_women_* = 0.800, *CI:* 0.717–0.892, *p* < 0.001) emerging as statistically significant independent predictors of ESR states. Having more depressive symptoms was associated with a statistically significant increased probability of being in ESR states among women (*OR_women_* = 1.063, *CI:* 1.017–1.111, *p* < 0.001) but not among men. Compared to women who are married and living with their spouse, women who were divorced had a statistically significant increased probability (*OR_women_* = 1.815, *CI:* 1.259–2.615, *p* = 0.001) of being in ESR states, a statistical effect that was not evident among males.

### 3.2 Gendered probability of feeling lonely in objective ESR states

The binary logistic model (Model 4) examining the probability of loneliness among older persons in ESR states yielded a statistically significant model [*χ*^2^(26) = 204.878, *p* < 0.001, Nagelkerke *R^2^* = 0.273]. Living alone (*OR* = 0.496, *CI:* 0.312–0.788, *p* = 0.003), more depressive symptoms (*OR* = 1.399, CI: 1.290–1.517, *p* < 0.001), decreased social participation (*OR* = 0.906, *CI:* 0.832–0.987, *p* = 0.024), as well as being less extraverted (*OR* = 0.828, *CI:* 0.695–0.985, *p* = 0.034) were statistically significantly associated with an increased probability of being lonely within ESR states. Compared to older persons in ESR states that are retired, being unemployed increased the probability (*OR* = 0.399, *CI:* 0.160–0.994, *p* = 0.048) of feeling lonely within ESR states (see Table 3).

**Table 3:** Binary logistic regression analyses (Models 4, 5, 6) predicting loneliness among older persons in objective states of exclusion from social relations (ESR), with demographics, EURO-D, health, social participation, and personality traits as predictors.

	Model 4 (all)		Model 5 (men)		Model 6 (women)	
	B	Exp(B)	B	Exp(B)	B	Exp(B)
Gender (male/female)	−0.155	0.856	–	–	–	–
Age	−0.004	0.996	0.005	1.005	−0.011	0.989
Education	0.030	1.030	0.019	1.020	0.056	1.058
Living alone (yes/no)	−0.701*	0.496	−1.013*	0.363	−0.646	0.524
Income	0.000*	1.000	0.000	1.000	0.000	1.000
Marital status^1^						
Registered partnership	−1.242	0.289	−20.56	0.000	0.598	1.819
Married not cohabiting	0.795	2.215	0.711	2.035	0.503	1.654
Never married	0.260	1.298	0.085	1.089	0.365	1.440
Divorced	0.042	1.043	−0.334	0.716	0.308	1.360
Widowed	0.314	1.368	0.323	1.381	0.412	1.509
Employment status^2^						
(Self) Employed	−0.122	0.885	−0.012	0.988	−0.284	0.752
Unemployed	−0.919*	0.399	−1.745*	0.175	0.192	1.212
Permanently sick	−0.278	0.757	−0.147	0.863	0.227	1.254
Homemaker	0.323	1.382	–	–	0.351	1.421
Other	−0.087	0.917	0.798	2.221	−0.407	0.665
ADL	0.030	1.030	−0.159	0.853	0.272	1.313
IADL	0.102	1.108	0.422	1.524	−0.138	0.871
Chronic diseases	0.064	1.066	0.001	1.001	0.152	1.164
Mobility limitations	−0.043	0.957	−0.035	0.966	−0.053	949
EURO-D	0.336*	1.399	0.336*	1.399	0.337*	1.400
Social participation	−0.098*	0.906	−0.068	0.056	−0.205*	0.815
Personality traits						
Extraversion	−0.189*	0.828	−0.087	0.917	−0.371*	0.690
Agreeableness	0.001	1.001	−0.261	0.771	0.275	1.317
Conscientiousness	−0.033	0.968	0.040	1.041	−0.124	0.883
Neuroticism	0.130	1.139	0.093	1.097	0.187	1.206
Openness	−0.008	0.992	−0.117	0.889	0.166	1.180

^1^Reference category: married and living with spouse. ^2^Reference category: retired. ADL, Activities of daily living; IADL, Instrumental activities of daily living; EURO-D, Depressive symptoms scale. Significance level: *p < 0.050. Statistically significant values at the *p* < 0.050 are in bold.

To examine gender differences in the prediction of loneliness among older persons in ESR states, the same model was used in gender stratified analyses excluding gender as a predictor (Models 5 and 6). The analyses yielded statistically significant model for both men [*χ^2^_men_* (24) = 103.430, *p* < 0.001, Nagelkerke *R^2^* = 0.271] and women [*χ^2^_women_* (25) = 482.356, *p* < 0.001, Nagelkerke *R^2^* = 0.330]. For both genders, having more depressive symptoms remained a statistically significant factors related with an increased probability of feeling lonely in ESR states (*OR_men_* = 1.399, *CI:* 1.233–1.558, *p* < 0.001; *OR_women_* = 1.400, *CI:* 1.251–1.567, *p* < 0.001). Living alone was statistically significant related with an increased probability of feeling lonely for men (*OR_men_* = 0.363, *CI:* 0.180–0.734, *p* = 0.005) but not for women. Less social participation (*OR_women_* = 0.815, *CI:* 0.703–0.944, *p* = 0.007) and less extraversion (*OR_women_* = 0.690, *CI:* 0.520–0.916, *p* = 0.010) was statistically significant related with an increased probability of feeling lonely within ESR states for women but not for men. Compared to men that are retired, being employed increased the probability of feeling lonely within ESR states (*OR_men_* = 0.175, *CI:* 0.041–0.738, *p* = 0.018). No statistical effect of employment status on loneliness was evident among older women within ESR states (see Table 3).

## 4 Discussion

This study was set to examine the situational (i.e., socioeconomic factors, living arrangements, marital and employment status, physical and mental health) and dispositional factors (i.e., personality traits) associated with different conjunctions of loneliness and ESR states (i.e., ESR and lonely, ESR and not lonely, not ESR but lonely) in older age. It was hypothesized that gender differences will emerge in the associations of situational factors and personality traits with the probability of being in ESR states at an older age. It was also hypothesized that among older persons that are challenged by ESR states, higher neuroticism and lower extraversion in women, and lower agreeableness in both genders will be associated with higher levels of loneliness.

The results indicate that over half (54%) of older persons that are in ESR states do not experience any loneliness. Previous research among older Europeans have indicated that older women have larger social networks then older men and are more likely to report network growth despite lower family involvement over time (34). Consistent with this trend, the results of this study indicate that the probability of ESR states in older age is 69% higher among older men than among older women. However, the assumption that older women have a greater ability to maintain social connections when facing ESR-related challenges received only partial support in this study. For both genders, living alone, never being married, being widowed, and being retired emerged as situational factors that substantially increase the probability of ESR states in older age (i.e., from 54 to 121%).

Confirming the study’s first hypothesis, divorce increased the probability of ESR states among older women but not among older men, possibly reflecting the decreased likelihood of women to re-partner after a potential marriage resolution in older age (36, 37). The fact that women live longer than men means that the re-partnering pool for heterosexual women shrinks in later life (37). However, many older women enjoy the autonomy and independence following marital dissolution and are reluctant to re-partner due to the fear of gendered expectations for caregiving (46, 47). Yet, notable gender differences are observed in this study in the value of social participation (i.e., activities within a community) in avoiding ESR states in older age, where decreased social participation was twice as a much stronger associated with ESR among older women compared to older men. This evidence suggests that socialization outside the immediate family can buffer against the risks associated with ESR states. Older women, in particular, seem to benefit more than men by maintaining social connections in “third places.”

The findings of this study indicate that less conscientiousness increases the probability of being in ESR states among older men (to 13%) but not among older women. Buecker et al. (22) argue that maintaining regular contact with friends and family can be regarded as a responsible and reliable behavior of a conscientious person. The cultural expectations for social affiliation assigned to the female population and the tendency of older women to maintain larger social networks compared to older men may render the issue of conscientiousness a less significant factor for their socialization later in life. In turn, an ESR state may be more likely among less conscientious men, for whom their poorer efforts in maintaining regular contact with a larger network of significant others in later life may result in each loss being a major risk.

Being less extraverted emerged in this study as a factor that increases the probability of ESR states among older women (to 25%) but not among older men. Extraverted individuals prefer to have social interactions and enjoy the company of others (21). Having an extraverted personality in older age may indicate a better ability to use and extend the existing social network when facing ESR-related challenges (e.g., loss of a spouse). In turn, the risk of widowhood and the difficulties of re-partnering after a relationship dissolution are greater among older women than among older men (Antonucci et al., 2001) (36, 37). Hence, the results of this study may indicate that older women who are more extraverted are in a better position to expand their social network and compensate for the loss of a significant other in their lives, and by that, to avoid an ESR state.

Adding to the findings of Litwin and Levinsky (24), the results of this study indicate that the positive associations of certain personality traits with an increased risk of ESR states in older age might be gendered. Yet, the results of the present study also suggest that personality traits are weaker correlates of ESR states than situational factors might be in older age. In other words, the evidence of this study suggests that social context may have a predominant role in shaping ESR states in older age.

Among older persons in ESR states, the significance of living alone in predicting the probability of feeling lonely seems to be also gendered. More precisely, older men in ESR states who lived alone were 2.75 times more likely to feel lonely than older men who were cohabiting. There was no evidence of an analogous risk among older women within ESR states. This is consistent with previous findings (29) showcasing that living alone is a strong predictive factor of loneliness among older Europeans, with the results of this study adding that living alone may have a more detrimental effect among older men than among older women. Echoing the relative importance of social participation in avoiding ESR states for older women, less social participation was associated with an increased probability (23%) of loneliness among older “network-less” women, with this association not statistically evident among older men. These findings are consistent with previous research (32) indicating observable benefits from an increased social participation among those with a deficient social network in older age. Adding to the current state of knowledge, the benefits of maintaining social connections in “third places” appear in this study to be more pronounced among older women that face ESR challenges.

Partially confirming the second hypothesis of this study, lower extraversion was associated with an 45% increase in the probability of feeling lonely for older women in ESR states. Deviating from the results of Schutter et al. (20), there was no evidence that higher neuroticism in women, and lower agreeableness in both genders was associated with loneliness among older persons living in ESR states. A potential explanation is that Schutter et al. (20) did not consider older persons in ESR states as a distinct category of the older population, as social network size was included in their models as a continuous variable. Thus, loneliness may be associated with neuroticism in older women, as well as with lower agreeableness in both genders, when the reduction of older person social network does not reach levels of social isolation at the extreme.

The assumption that an increased depressive symptomatology in older age contributes to social withdrawal received only weak support in the present study. More precisely, having more depressive symptoms was associated with a slight increase (6%) to the probability of ESR states only among older women but not among older men. However, an increased number of depressive symptoms was associated with a substantial increase (i.e., to 40%) in the probability of feeling lonely among older persons in ESR states. This evidence echoes the overlapping nature of loneliness and depression (48), suggesting that an increased depressive symptomatology is related to loneliness among older persons that are challenged by ESR states, irrespective of their gender.

The findings of this study should be considered within the limitations and restrictions of a cross-sectional design, including the inability to interpret causational associations between situational or dispositional factors with the probability of ESR states in older age. An additional limitation in this study stems from the brevity of the Big Five personality measure, which has a poor internal consistency and may have not allowed to capture the full breadth of associations between personality and sociability in older age. While living alone and certain marital statuses indicate a lack of co-habitation (i.e., never being married, being widowed, divorced), it is possible that certain personality traits can predict both a preference to live alone and the inclination to refrain from a stable romantic partnership. Importantly, ESR states in this study did not signify isolation from any form of human contact, as the social network inventory used in SHARE asks mainly about older adults’ confidants; thus, most probably close relations in older age. The participants’ social participation in “third places,” namely outside the core family and work environments, most probably refers to social connections that are less significant; although the measure of social participation in this study lacks precision in this respect.

## 5 Conclusion

As a conclusion, the probability of ESR states is higher among older men than among older women, with retirement and circumstances indicating a lack of co-habitation (i.e., living alone, never being married, being widowed) to constitute situational factors that are substantially associated with the probability of ESR in older age. Among the notable gender differences is that of an eventual divorce, which was associated with an increased probability of ESR states among older women but not among older men. The significance of personality traits in predisposing older persons to a solitary life was supported only to some extent, with less extraverted women and less conscientious men to be more likely to be situated in ESR states. Overall, the results of the study suggest that personality traits are weaker associated with ESR states than situational factors, hence that social context may have a predominant role in shaping ESR states in older age. While the probability of loneliness to typify ESR states seems proportionally leveled between the genders, only older women that are challenged by both ESR states and loneliness tend to also report increased depressive symptomatology. Living alone seems to be associated with loneliness among older men in ESR states, whereas older women in ESR states are more likely to feel lonely if they are less participatory in social activities, or if they are less extraverted. It is concluded that a gender perspective is necessary when assessing the risks of ESR, loneliness, and their conjoint conditions in older age, as different situational and dispositional factors are associated with different risks to the wellbeing of older persons.

## Data Availability

The data analyzed in this study is subject to the following licenses/restrictions: The data that support the findings of this study are available from the Survey on Health, Aging, and Retirement in Europe SHARE, but restrictions apply to the availability of these data, which were used under license for the current study, and so are not publicly available. Data are, however, available from SHARE BERLIN Institute GmbH (SBI) upon reasonable request and with permission of this organization. The SPSS syntax file leading to the results of this study is available from the corresponding author upon reasonable request. Requests to access these datasets should be directed to Share Berlin Institute GmbH (SBI), info@share-project.org.

## Footnotes

^1^ https://share-eric.eu/data/data-documentation/methodology-volumes
